# Bridging Therapies With Injectable Immunomodulatory Drugs in the Management of Multiple Sclerosis: A Delphi Survey of an Italian Expert Panel of Neurologists

**DOI:** 10.3389/fneur.2022.898741

**Published:** 2022-07-15

**Authors:** Girolama Alessandra Marfia, Diego Centonze, Marco Salvetti, Elisabetta Ferraro, Valentina Panetta, Claudio Gasperini, Massimiliano Mirabella, Antonella Conte

**Affiliations:** ^1^Multiple Sclerosis Clinical and Research Unit, Department of Systems Medicine, Tor Vergata University, Rome, Italy; ^2^Department of Systems Medicine, Tor Vergata University, Rome, Italy; ^3^Unit of Neurology, IRCCS Neuromed, Pozzilli, Italy; ^4^Department of Neuroscience, Mental Health, and Sensory Organs, Sapienza University of Rome, Rome, Italy; ^5^ASL Rome1 P.O. San Filippo Neri U.O.C Neurologia -Centro Sclerosi Multipla, Rome, Italy; ^6^L'AltraStatistica srl – Consultancy & Training, Biostatistics Office, Rome, Italy; ^7^Department of Neuroscience, S. Camillo Forlanini Hospital, Rome, Italy; ^8^Multiple Sclerosis Center, Fondazione Policlinico Universitario “A. Gemelli” IRCCS, Rome, Italy; ^9^Department of Neurosciences, Centro di Ricerca Sclerosi Multipla (CERSM), Università Cattolica del Sacro Cuore, Rome, Italy; ^10^Department of Human Neuroscience, Sapienza University of Rome, Rome, Italy; ^11^IRCCS Neuromed, Pozzilli, Italy

**Keywords:** multiple sclerosis, bridging therapy, Delphi survey, MS management, injectable immunomodulatory drugs

## Abstract

**Background:**

In multiple sclerosis (MS), bridging therapies are usually administered when switching from one therapy to another. Such treatments generally consist of injectable immunomodulatory drugs (interferon or glatiramer acetate), whose efficacy, safety, and tolerability data are consolidated for use even in fragile patients. We performed a nationwide survey to gather expert opinions regarding the most appropriate use of bridging therapies in MS.

**Methods:**

An independent steering committee of Italian neurologists with expertise in MS treatment identified critical issues in the use of bridging therapies and formulated a questionnaire. This questionnaire was used to conduct a Delphi web survey, involving a panel of Italian neurologists with experience in MS treatment. Their anonymous opinions were collected in three sequential rounds. Consensus was defined as an interquartile range (IQR) ≤2.

**Results:**

Responses were obtained from 38 experts (100%) in all three rounds. Injectable immunomodulatory drugs were considered first-line therapy in patients with mild-to-moderate disease activity and in women planning to become pregnant. In addition, the experts were confident about prescribing these drugs in patients at risk of cancer recurrence, while the panel agreed to discontinue any treatments in patients with uncontrolled cardiovascular or metabolic disorders. Moreover, bridging therapy with injectable immunomodulatory drugs was considered appropriate in order to protect the patient from disease reactivation when a prolonged washout was needed and also while waiting for the completion of the immunization schedule.

**Conclusion:**

The results of this nationwide survey confirm that, among Italian neurologists, there was wide agreement on the use of bridging therapies with injectable immunomodulatory drugs in several conditions in order to minimize the risk of disease reactivation when a prolonged washout was required or when the immunization schedule still needed to be completed in patients planning to become pregnant and in patients at risk of cancer recurrence.

## Introduction

The term “bridging therapy” is used in medicine to indicate a transitional period to another stage of therapy or health. This concept is well-known and widely applicable in the field of transplantation (1, 2) and anticoagulant treatment (e.g., heparin bridge) (3). Therapeutic plasma exchange and intravenous immunoglobulins are examples of rapid but short-acting immunomodulatory treatments used as a bridge while waiting for slower-acting immunosuppressive therapies to become effective in other autoimmune neurologic diseases, such as myasthenia gravis (particularly when glucocorticoid use has to be avoided or minimized).

In multiple sclerosis (MS), bridging therapies may be administered when switching from one therapy to another. Such treatments generally consist of injectable immunomodulatory drugs (interferon or glatiramer acetate), whose efficacy, safety, and tolerability data are consolidated for use even in fragile patients. In the past, monthly pulses of intravenous steroids were suggested as an option to prevent reactivation of MS in subjects switching from natalizumab to alemtuzumab or in patients discontinuing fingolimod (4). Moreover, if the chosen disease-modifying treatment (DMT) could not be administered immediately, due, for example, to persistent leukopenia, a bridging therapy with corticosteroids, interferons, or glatiramer acetate was considered a valid option to fill this treatment gap.

However, while the concept of bridging therapy in MS is relatively new and still not adequately defined in terms of duration, it still might play an important role in MS decision-making strategies. In 2019, interferon labeling was updated to indicate that it could be safely used during pregnancy and breastfeeding, suggesting its potential role as a bridging treatment in female patients with MS with mild disease activity who plan on becoming pregnant in the short term (5–7).

The aim of this survey was to obtain expert opinions on the use of bridging therapies with injectables in MS from 38 Italian neurologists highly qualified in treating MS.

## Materials and Methods

An independent steering committee of seven Italian neurologists with expertise in the treatment of MS identified critical issues concerning bridging therapies and generated a 16-item questionnaire.

This questionnaire was used to conduct a Delphi web survey with an expert panel consisting of 38 neurologists from 25 Italian MS centers.

The Delphi technique is considered an effective way to gain and measure group agreement in healthcare consensus development methods (8). It is an anonymous structured approach that uses repeated administration (rounds) of the same questionnaire given to a panel of experts (8, 9). Anonymity can reduce the effects of status, personality, and group pressure that can arise in meetings and can help resolve several difficulties typically due to group decision dynamics. Questionnaire items are provided by a small group of experts, called the board, and submitted to the entire panel. During the following rounds, the administrator who manages the process, called the facilitator, provides participants with a statistical summary of the responses from all respondents from the previous round and invites the experts to provide reasons if there is no consensus of opinion (9).

Three consensus rounds were executed over nearly 5 months (from December 2019 to April 2020). All responses were aggregated to maintain respondent anonymity. Review and approval of this study by an ethics committee were not necessary since the collected data consisted of neurologist opinions. In each round, the participants were invited to respond by scaling each statement based on the degree of agreement (ranging from 1 = no agreement to 7 = maximum agreement).

The interquartile range (IQR) was used as a measure of the deviation of the individual expert's opinion from the opinion of the whole panel (median value). The IQR is the difference between the 3rd and 1st quartile in which the middle 50% of evaluations were located.

Consensus was defined as an IQR ≤2 and agreement with the statement when the 1st quartile was ≥4. For all 16 questions, the following statistical parameters were calculated: median, 1st and 3rd quartile, and IQR. Stata 16.1 was used for all analyses and graphs.

## Results

Responses were obtained from 38 experts (100%) in all three rounds. Between the second and third rounds, 39% and 23% of the respondents changed their responses, respectively. All statements are shown in Table 1.

**Table 1:** A Delphi questionnaire.

1. The onset of drug action plays a key role in choosing bridging treatment. 2. At diagnosis, I administer injectable immunomodulatory drugs in patients with mild-to-moderate disease activity and in women who wish to become pregnant in the short term. 3. Clinical evidence regarding the safety profile of interferon beta and glatiramer acetate during pregnancy is strong. 4. Clinical evidence regarding the safety profile of interferon beta during breastfeeding is strong. 5. I prescribe an approved immunomodulatory therapy during pregnancy. 6. I prescribe an approved immunomodulatory therapy during breastfeeding. 7. Clinical evidence regarding the safety profile of injectable immunomodulatory drugs on cancer risk is strong. 8. In patients with MS with a history of previous cancer, I prescribe an injectable immunomodulatory therapy. 9. In patients with MS with uncontrolled cardiovascular and metabolic diseases, I discontinue any treatment. 10. I perform an extended infection risk assessment at the time of diagnosis. 11. I perform an extended infection risk assessment only when switching to second-line therapies. 12. I performan extended infection risk assessment only when patients are therapy free. 13. During the infection risk assessment, it is important to prescribe injectable immunomodulatory drugs to protect the patient from disease reactivation. 14. While waiting for the immunization schedule to be completed, bridging therapy with injectable immunomodulatory drugs is appropriate. 15. I use injectable immunomodulatory drugs in patients with a not-yet-well-defined prognosis due to pending clinical or instrumental data or a short temporal window from the disease onset. 16. When switching MS treatments, I minimize the risks associated with a prolonged washout by administering bridging therapies.

**Figure 1:**
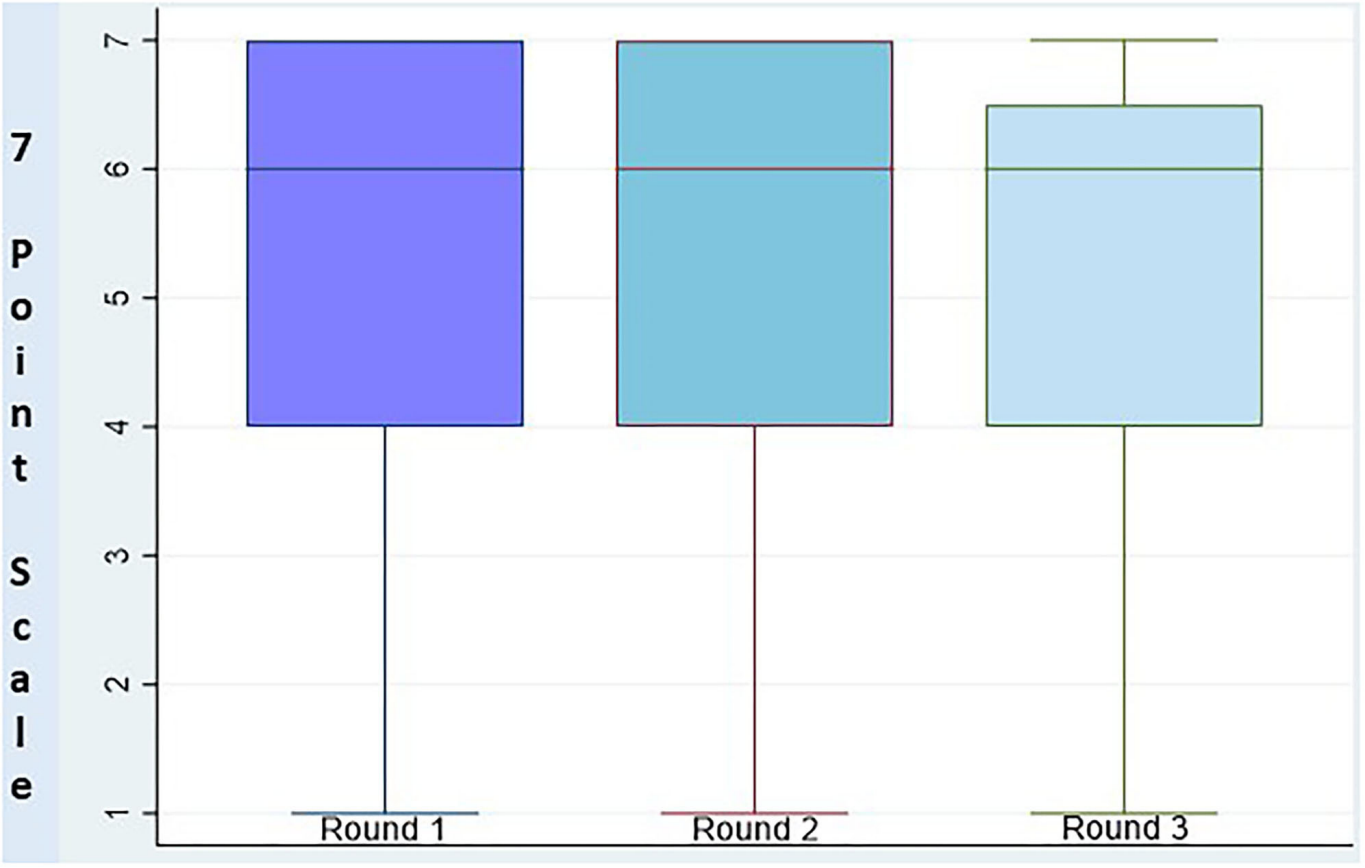
Distribution of responses between rounds.

**Figure 2:**
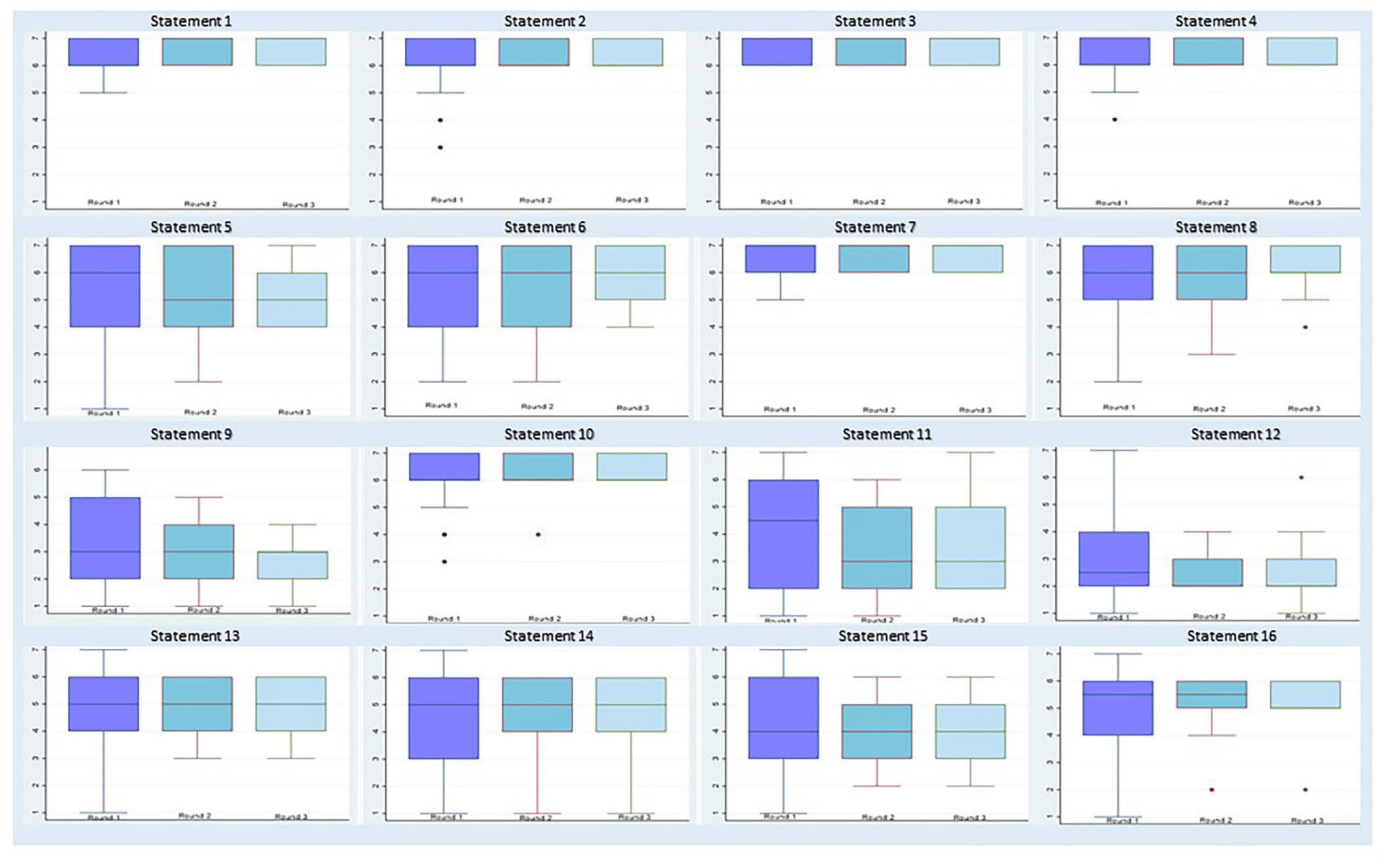
Distribution of responses for each item per round.

High positive consensus was obtained for12 statements, while two statements reached a negative consensus (Items 9 and 12). In one case, the panel disagreed with the statement but did not reach a consensus (Item 11), and, in another case, there was indecision regarding the statement (Item 15; Figures 1, 2, Supplementary Figure S1).

The respondents stated that the time necessary for the onset of drug activity played a critical role in choosing a bridging therapy. At the time of diagnosis, injectable immunomodulatory drugs were confirmed to be the first choice in patients with mild-to-moderate disease activity and in women who were planning to become pregnant in the short term. Neurologists agreed that scientific evidence supporting the safety of interferon and glatiramer acetate administration during pregnancy was robust, although the label of glatiramer acetate suggested avoiding its use unless the benefits outweighed the risks. The neurologists also agreed that scientific evidence regarding interferon use during breastfeeding was robust. In clinical practice, they prescribed immunomodulatory treatments approved for pregnancy and breastfeeding in patients who were pregnant or breastfeeding. Moreover, all experts were confident about prescribing injectable immunomodulatory drugs in patients at risk of cancer recurrence.

The respondents stated that they discontinued any immunomodulatory treatment in patients with uncontrolled cardiovascular or metabolic disease.

There was agreement on the statement that an extensive infection risk assessment should be performed at the time of diagnosis. However, a consensus was not reached when they were asked if they actually performed this extensive assessment before switching to second-line therapies (Item 11). It was agreed that an extended infection risk assessment should be performed only in immunosuppressive drug-free patients to avoid the risk of latent infection reactivation and interference with laboratory tests.

During the evaluation of infection risk, the experts highlighted the critical issue of protecting patients from disease reactivation by administering injectable immunomodulatory drugs as a bridging therapy. This behavior was considered appropriate also while waiting for the immunization schedule to be completed.

Item 15 resulted in indecision among neurologic health professionals regarding the use of injectable immunomodulatory drugs in patients with a not-yet-well-defined prognosis due to pending clinical findings and/or instrumental assessment or a short temporal window from the disease onset.

Regarding switching from one DMT to another, the neurologists were in favor of using a bridging therapy in order to minimize the risk of disease reactivation when prolonged washout was required in individual patients. When the various items were discussed, it was clearly intended that bridging therapy duration would outlast the 8–12 weeks required for injectables to be effective (10, 11).

## Discussion

The objective of this Delphi analysis was to obtain consensus on the choice and most appropriate use of bridging therapy in MS. In summary, 14 statements achieved a consensus in the survey. There was positive consensus on 12 statements and negative consensus on two statements.

A rapid onset of action was confirmed to be a critical issue driving the choice of bridging treatment, and this approach may play a key role during the current pandemic period. Interferon beta does not increase the risk linked to SARS-CoV-2, and, indeed, some studies have highlighted the protective effect of this drug as indicated as a potential antiviral treatment of coronavirus-related diseases (COVID-19, MERS, and SARS) (12–17). According to literature data, Italian neurologists participating in this survey consider interferon and glatiramer acetate as first-line treatment in patients with mild-to-moderate disease activity at early stages (18). Although there are no evidence-based guidelines on decision-making in family planning, these first-line treatments are considered appropriate strategies in women with MS who desire to become pregnant in the short term (19).

Until a few years ago, clinical treatment guidelines recommended that injectables, such as interferon be discontinued at pregnancy occurrence (20, 21). However, interferons are now considered safe in pregnancy and have obtained approval for use during pregnancy in Europe (5–7). Moreover, all injectables are no longer contraindicated during breastfeeding according to the recent label updates (2019 for interferons and 2022 for glatiramer acetate). This modified prescription label now allows interferons to be recommended from conception, during the whole gestational period, and while breastfeeding (22). Therefore, a switch to interferon may be considered for female patients with MS on oral first-line DMTs that need to be discontinued due to pregnancy planning (i.e., dimethylfumarate, teriflunomide).

In regard to currently available DMTs, several of which have immunosuppressive effects, screening patients with MS for potential malignancy risk has become crucial, especially in older patients in whom comorbidity risk is higher. Since interferon and glatiramer acetate are considered to have a favorable and well-documented safety profile and were not associated with cancer in clinical trials (23), they tend to be preferred in patients with MS with comorbidities and, in particular, in people at risk of cancer or cancer recurrence. Some disorders, including uncontrolled cardiovascular and metabolic diseases, remain a critical issue and neurologists are less confident in prescribing even injectable DMTs in these conditions due to the perceived overall benefit-risk ratio.

According to prescription label recommendations, screening for chronic infections (e.g., hepatitis B and C, tuberculosis) is required before initiating specific DMTs. Patients who test positive for latent infections must be treated before starting these drugs. In the last few years, however, an extended infection risk assessment has been widely recommended regardless of the DMT product label. To avoid possible false-negative results due to the interference of immunosuppressive drugs, this assessment should be performed in therapy-free patients. Moreover, an extensive infection risk assessment performed at the time of diagnosis in naïve patients may avoid delays in switching to a second-line treatment during the disease course and may help to identify potential subclinical comorbidities. This beneficial approach, however, is not always applied in clinical practice. In light of these considerations, prescribing a bridging therapy with injectable immunomodulatory drugs (with a slightly prevalent use of high-dosage subcutaneous interferon beta) may protect patients from disease reactivation during the evaluation of infection risk or while waiting to complete the immunization schedule, thus minimizing the risks associated with a prolonged washout. Although not detailed, it is worth noting that, for all clinical conditions considered in the Delphi panel, the time interval intended to be covered by bridging therapy outlasted the known interval required for the injectables to be active as DMTs (i.e., longer than 2–3 months).

A limitation of this study is related to the Delphi technique itself; in particular, the opinions reported are those of a select group of experts from a few Italian centers, and their approach may not be representative of Italian neurologists and clinical practice in other countries. Another limitation is related to the type of bridging drugs investigated. We specifically considered bridging with injectables and not bridging when switching from some second-line therapies to prevent rebound or bridging with natalizumab in patients on second-line DMTs in case of pregnancy desire. Thus, expert consensus is still needed regarding the unaddressed bridging of second-line DMTs. More importantly, the present study only evaluated the potential role of injectables used as bridging therapy in specific clinical conditions according to MS neurologists, but it did not address their effectiveness as bridging therapy. Nonetheless, the present Delphi study paves the way toward future clinical studies specifically designed to assess the effectiveness of injectables as bridging therapy for the various clinical conditions identified by the MS expert panel. To our knowledge, this is the first survey based on a panel of experts (neurologists) that has tried to obtain consensus on the use of bridging therapy with injectables in MS management.

## Conclusions

The results of this nationwide survey confirm that Italian neurologists agree on the use of bridging therapy with injectable immunomodulatory drugs in several conditions in order to minimize the risk of disease reactivation when a prolonged washout is required or the immunization schedule still needs to be completed in patients who plan on becoming pregnant and in patients at risk of cancer recurrence.

## Data Availability Statement

The raw data supporting the conclusions of this article will be made available by the authors, without undue reservation.

## Author Contributions

VP performed the statistical analysis. GM, MM, and AC wrote the first draft of the manuscript. DC, MS, CG, and EF wrote sections of the manuscript. All authors contributed to conception and design of the study, contributed to manuscript revision, read, and approved the submitted version.

## Funding

The development of this publication was financially supported by Merck Serono Italy, an affiliate of Merck KGaA, Darmstadt, Germany, through an independent medical writing grant. The funder was not involved in the study design, collection, analysis, interpretation of data, the writing of this article or the decision to submit it for publication.

## Author Disclaimer

The views and opinions described in this publication do not necessarily reflect those of the grantor.

## Conflict of Interest

GM received honoraria for speaking, consultation fees, and travel funding from Roche, Almirall, Bayer Schering, Biogen Idec, Merck Serono, Novartis, Sanofi-Genzyme, Mylan, and Teva. She is the principal investigator in clinical trials for Actelion, Biogen Idec, Merck Serono, Mitsubishi, Novartis, Roche, Sanofi-Genzyme, and Teva. DC is an Advisory Board member of Almirall, Bayer Schering, Biogen, GW Pharmaceuticals, Merck Serono, Novartis, Roche, Sanofi-Genzyme, and Teva and received honoraria for speaking or consultation fees from Almirall, Bayer Schering, Biogen, GW Pharmaceuticals, Merck Serono, Novartis, Roche, Sanofi-Genzyme, and Teva. He is also the principal investigator in clinical trials for Bayer Schering, Biogen, Merck Serono, Mitsubishi, Novartis, Roche, Sanofi-Genzyme, and Teva. His preclinical and clinical research was supported by grants from Bayer Schering, Biogen Idec, Celgene, Merck Serono, Novartis, Roche, Sanofi-Genzyme, and Teva. MS reports speaking honoraria and research support from Merck, Sanofi, Novartis, Biogen, Roche, Bristol Myers Squibb. EF has received travel grants from Biogen, Merck, Novartis, Sanofi-Genzyme, and Roche. VP is employed by L'altrastatistica srl – Consultancy & Training, Rome, Italy. CG received a fee as speaker or advisory board by Teva, Novartis, Roche, Merck KGaA, Bayer, Almirall, and Biogen. MM is a scientific advisory board membership of Bayer Schering, Biogen, Sanofi-Genzyme, Merck, Novartis, Teva, Mylan, Almirall. He received consulting and/or speaking fees, research support or travel grants from Almirall, Bayer Schering, Biogen, CSL Behring, Sanofi-Genzyme, Merck, Novartis, Teva, Roche, and Ultragenix. He is principal investigator in clinical trials for Biogen, Merck, Novartis, Roche, Sanofi Genzyme, Teva, Ultragenix, and CSL Behring. AC received research grants or speaking honoraria and board participation from Almirall, Biogen, BMS-Celgene Merck, Novartis, Roche, Sanofi-Genzyme.

## Publisher's Note

All claims expressed in this article are solely those of the authors and do not necessarily represent those of their affiliated organizations, or those of the publisher, the editors and the reviewers. Any product that may be evaluated in this article, or claim that may be made by its manufacturer, is not guaranteed or endorsed by the publisher.

## Supplementary Material

Supplementary Figure S1 Data for each item per round.
