# Supplementary figures and images for: Bridging Therapies With Injectable Immunomodulatory Drugs in the Management of Multiple Sclerosis: A Delphi Survey of an Italian Expert Panel of Neurologists
Source: Front Neurol. 2022 Jul 15;13:898741. doi: 10.3389/fneur.2022.898741 (PMC9337240; doi:10.3389/fneur.2022.898741)

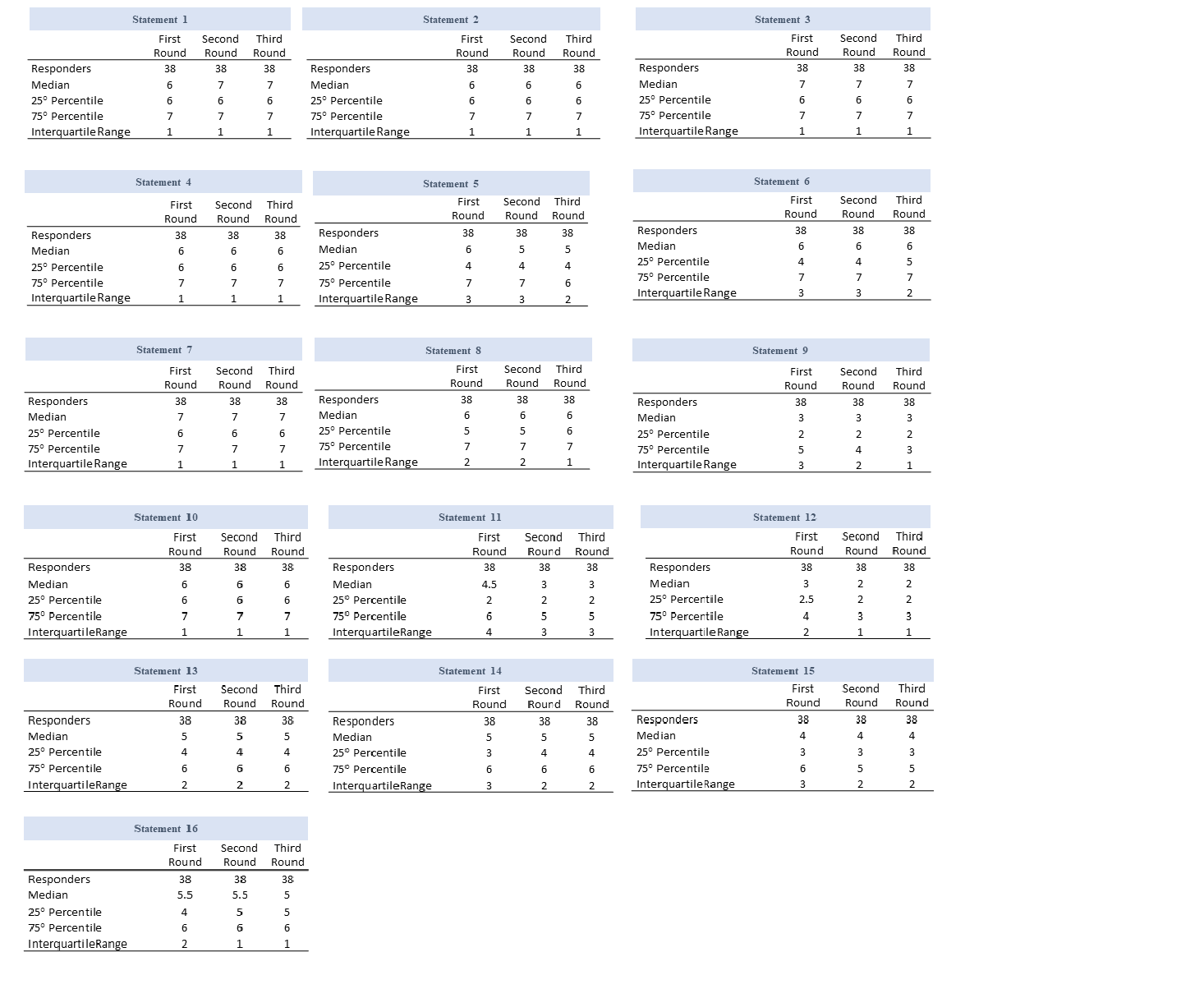

Supplement: Supplementary Figure S1 — Data for each item per round. [file Image_1.PNG]
